# Immunotherapy in advanced biliary tract cancer: current status and future directions

**DOI:** 10.3389/fphar.2026.1927523

**Published:** 2026-09-07

**Authors:** Wu Lv, Zhengjia Liu, Hui Du, Guilin Yu, Wen Tan, Xiaoquan Yang

**Affiliations:** 1 Department of International Medical Services (Surgical Ward), Cancer Hospital of Dalian University of Technology, Liaoning Cancer Hospital and Institute, Shenyang, China; 2 Department of Radiology, The Fourth Affiliated Hospital of China Medical University, Shenyang, China; 3 Department of Orthopedics, Fuxin Traditional Chinese Medicine Hospital, Fuxin, China; 4 Department of Surgery, Donggang Hospital of Traditional Chinese Medicine, Donggang, China

**Keywords:** adoptive cell therapy, biliary tract cancers, chemoimmunotherapy, immune checkpoint inhibitors, predictive biomarkers, tumor microenvironment

## Abstract

Biliary tract cancers (BTCs) are aggressive, molecularly heterogeneous malignancies, and most patients present with unresectable or metastatic disease at diagnosis. For more than a decade, gemcitabine–platinum chemotherapy served as the first-line standard of care, with only modest survival benefit. The phase 3 TOPAZ-1 and KEYNOTE-966 trials have now established chemoimmunotherapy with immune checkpoint inhibitors as the new standard front-line regimen, and the phase 2 IMbrave151 trial is evaluating whether adding antiangiogenic therapy provides further benefit. Nonetheless, only a minority of patients derive durable clinical benefit from immunotherapy. Conventional predictive biomarkers—deficient mismatch repair or high microsatellite instability, tumor mutational burden, and PD-L1 expression—have limited discriminatory value in BTC, largely because of profound intratumoral heterogeneity. Emerging biomarkers and composite multi-feature models show greater promise for refining patient stratification. Investigational modalities, including therapeutic cancer vaccines, chimeric antigen receptor T cells, and tumor-infiltrating lymphocyte therapy, have shown preliminary antitumor activity but require validation in larger cohorts. Multiple combination regimens aimed at reversing the immunosuppressive tumor microenvironment are under active investigation, and disease-specific protocols for managing hepatobiliary immune-related adverse events remain an unmet clinical need. In this review, we synthesize the latest evidence on biomarkers, clinical trials, combination strategies, cellular therapies, and safety management for immunotherapy in advanced BTC, and we highlight persistent challenges and future research directions.

## 1 Introduction

Biliary tract cancers arise from the biliary epithelium and comprise three major anatomic subtypes: intrahepatic cholangiocarcinoma (ICC), extrahepatic cholangiocarcinoma (ECC), and gallbladder cancer (GBC) (Banales et al., 2020). Each subtype has a distinct molecular landscape: ICC is characterized by frequent IDH1/2 mutations, FGFR2 fusions, and ARID1A alterations (Nakamura et al., 2015); ECC is enriched for KRAS, SMAD4, and TP53 mutations (Valle et al., 2021); and GBC has the highest prevalence of ERBB2 (HER2) amplification (Javle et al., 2021). Globally, more than 210,000 new cases are diagnosed annually, with marked geographic variation driven by region-specific risk factors—liver fluke infection in Southeast Asia, for example, and primary sclerosing cholangitis in Western populations (Khan et al., 2019). Five-year survival among patients with advanced disease remains below 20% (Kam et al., 2021). Only 30%–40% of patients present with disease amenable to curative-intent surgery, and even after complete resection, recurrence rates are high and long-term outcomes remain poor. For more than a decade, gemcitabine plus a platinum agent constituted the standard first-line systemic therapy, yielding a median overall survival of approximately 12 months (Valle et al., 2010). In the second-line setting, the ABC-06 trial established mFOLFOX as the reference regimen, but with a median overall survival of only 6.2 months, the gain was marginal, underscoring the substantial unmet need for more effective treatments (Lamarca et al., 2021). Over the past 5 years, immunotherapy—and immune checkpoint inhibition in particular—has produced durable responses in a subset of patients with BTC, paralleling its activity in other solid tumors. In this review, we synthesize biomarker and clinical trial data, evaluate combination strategies and emerging therapeutic modalities, and outline current advances, unresolved challenges, and future directions for immunotherapy in advanced BTC.

This narrative review draws on a literature search of PubMed, Web of Science, Embase, and the Cochrane Library from database inception through May 2026. Search terms and Medical Subject Headings (MeSH) paired BTC subtypes (cholangiocarcinoma, gallbladder cancer) with immunotherapeutic modalities (immune checkpoint inhibitors, adoptive cell therapy, cancer vaccines). We also manually searched reference lists of identified articles and conference proceedings from the American Society of Clinical Oncology and European Society for Medical Oncology. Eligible sources included peer-reviewed original research, clinical trials, meta-analyses, and reviews published in English that reported clinical, biomarker, or translational data on immunotherapy for advanced BTC. Editorials, case reports, and purely preclinical studies were excluded. After deduplication with EndNote software, 920 records were screened by title and abstract, 210 full-text articles were assessed for eligibility, and 54 studies were included in the final synthesis. Two authors independently screened and selected studies, with disagreements resolved by a third reviewer. Given the heterogeneity of study designs, study quality was appraised qualitatively on the basis of trial design, sample size, robustness of endpoints, and validation methodology.

## 2 Clinical biomarkers for immune checkpoint inhibitors

### 2.1 Deficient mismatch repair and microsatellite instability

Somatic mutations give rise to immunogenic neoantigens that shape tumor responsiveness to checkpoint inhibitors (Schumacher and Schreiber, 2015). Deficient mismatch repair (dMMR), caused by functional inactivation of DNA mismatch repair genes, impairs correction of replication errors; this mechanism is distinct from homologous recombination deficiency driven by BRCA1/2 or ATM alterations (Valle et al., 2017). In 2017, the U.S. Food and Drug Administration granted accelerated approval to pembrolizumab for previously treated solid tumors with dMMR or high microsatellite instability (MSI-H), including BTC—the first tissue-agnostic approval in oncology (Marcus et al., 2019). Clinical data consistently show robust responses to checkpoint inhibition in dMMR BTC. In a pooled analysis of five trials enrolling 149 patients with dMMR solid tumors, 11 had BTC; the objective response rate (ORR) in this subgroup was 27%, with response durations ranging from 11.6 to 19.6 months (Marabelle et al., 2020a). In the KEYNOTE-016 study, PD-1 blockade produced radiographic responses in 53% of patients with dMMR advanced cancers across 12 tumor types, with complete responses in 21% (Le et al., 2017). In the KEYNOTE-158 basket trial, 9 of 94 patients with dMMR tumors had BTC; the ORR was 37%, and median duration of response had not been reached at data cutoff (Marabelle et al., 2020a). These findings establish dMMR–MSI-H as the most robust predictive biomarker for checkpoint inhibition in BTC to date. Combinations of checkpoint inhibitors with DNA repair inhibitors and other immunomodulatory agents are currently under investigation. In a cohort of 321 BTC specimens, DNA repair gene alterations were detected in 13% of ICCs, 26% of ECCs, and 6% of GBCs (Javle et al., 2017). Even so, dMMR–MSI-H is rare in BTC overall, with prevalence varying widely across anatomic subtypes. Detection requires immunohistochemical analysis of mismatch repair proteins (MLH1, MSH2, MSH6, PMS2) paired with molecular testing (polymerase chain reaction or next-generation sequencing) for MSI (Greten et al., 2023). Assay standardization remains a critical issue, and at present, this biomarker guides treatment for only a very small fraction of patients.

### 2.2 Tumor mutational burden

High tumor mutational burden (TMB) is associated with checkpoint inhibitor efficacy across multiple solid tumor types. In a pan-cancer analysis, 82.1% of MSI-H tumors were classified as TMB-high, but only 18.3% of TMB-high tumors were MSI-H, indicating that TMB captures a broader population of immunogenic tumors (Marabelle et al., 2020b). Whole-exome sequencing of 231 BTCs by Nakamura and colleagues identified ultra-hypermutation—the highest TMB stratum—in approximately 6% of cases (7% of ICCs, 3% of ECCs, and 7% of GBCs) (Nakamura et al., 2015). Roughly 36% of ultra-hypermutated BTCs also harbor dMMR or MSI-H; these tumors carry abundant neoantigens and enriched gene sets encoding inhibitory checkpoint molecules (Mandal et al., 2019). Two fundamental, unresolved issues undermine the clinical utility of TMB in BTC: inconsistent threshold definitions and variable assay performance. No consensus exists on a BTC-specific cutoff for “high TMB.” The widely adopted pan-cancer threshold of 10 mutations per megabase (mut/Mb) classifies approximately 5%–8% of BTCs as TMB-high, but retrospective analyses show that most checkpoint inhibitor responses are concentrated in the ultra-hypermutated subgroup (>20 mut/Mb), which accounts for only 2%–3% of all BTCs (Javle et al., 2020). Tumors with intermediate TMB (5–19 mut/Mb) have response rates similar to those of TMB-low tumors, raising questions about whether the 10 mut/Mb cutoff has clinically meaningful predictive value in unselected BTC populations (McGrail et al., 2021). Second, TMB estimates are highly sensitive to assay design. Large-panel next-generation sequencing assays (≥300 genes) show moderate correlation with whole-exome sequencing, but small targeted panels systematically underestimate TMB and misclassify up to 30% of BTC cases (Chalmers et al., 2017). Concordance between tissue TMB and circulating tumor DNA (ctDNA)-based TMB is approximately 70% in BTC; discordance stems from spatial tumor heterogeneity, low ctDNA shedding, and artifacts from clonal hematopoiesis of indeterminate potential in liquid biopsies (Chalmers et al., 2017). These methodological inconsistencies mean TMB results from different laboratories are largely noninterchangeable, limiting their reliability for routine clinical decision making. Ongoing trials such as CheckMate 848 (NCT03668119) are comparing nivolumab plus ipilimumab with nivolumab monotherapy in solid tumors with high TMB (He et al., 2023). Preliminary 2025 data from the BTC subcohort (n = 42) showed a higher ORR with combination therapy (28.6%) than with monotherapy (14.3%); final overall survival data are pending (He et al., 2023).

### 2.3 PD-L1 expression

PD-L1 expression has been extensively evaluated as a predictive biomarker in BTC, but published findings are highly inconsistent. Reported positivity rates range from 4% to 76%; a 2025 meta-analysis of 56 studies involving 7,768 patients yielded a pooled positivity rate of 30%, with substantial variation according to antibody clone (58% for 5H1 vs. 17% for SP142, P = 0.02) and study design (48% in clinical trials vs. 26% in observational studies, P < 0.01) (Charoenngam et al., 2025). PD-L1 expression is generally higher in MSI-positive than in MSI-stable tumors, though one study found no such elevation in MSI-positive gallbladder cancers (Greten et al., 2023). Some reports suggest that higher PD-1 or PD-L1 expression predicts greater benefit from checkpoint inhibitors, but this finding has not been consistently replicated. In KEYNOTE-158, which evaluated pembrolizumab monotherapy in 104 patients with BTC, the ORR was only 5.8%, with no significant difference between PD-L1–positive (combined positive score [CPS] ≥1, 6.6%) and PD-L1–negative (2.9%) groups (Piha-Paul et al., 2020). These conflicting data arise from three layers of technical and biological variation. At the analytical level, antibody clones differ fundamentally in their target epitopes: clones 22C3 and 28–8 bind the intracellular C-terminal domain of PD-L1 and preferentially stain tumor cells, whereas SP142 recognizes an extracellular epitope and is far more sensitive to PD-L1 on immune cells, which explains its consistently lower tumor-cell positivity rates (Herbst et al., 2014). Even among assays targeting similar epitopes, overall interassay agreement can be as low as 64%–69% in BTC tissue because of differences in staining protocols and threshold calibration (Torlakovic et al., 2020). Scoring systems also differ: the tumor proportion score (TPS) assesses only tumor cells, whereas the CPS includes both tumor and immune cells, and the two metrics are not interchangeable (Topalian et al., 2012). A meta-analysis of phase 3 trials across cancer types suggested that a CPS cutoff of 1 or higher may be superior to TPS-based cutoffs for identifying patients unlikely to benefit, but BTC-specific validation is lacking (Sharma et al., 2021). A second source of variability is the profound intratumoral spatial heterogeneity of BTC. PD-L1 expression is often focal, concentrated at the invasive margin or around periductal inflammatory infiltrates, and can vary by an order of magnitude between different regions of the same tumor. A single core-needle biopsy samples only a tiny fraction of the tumor volume and carries a high risk of sampling bias, particularly in highly desmoplastic ICC with scattered tumor-cell nests. Discordance between primary tumors and metastatic sites is also well documented; hepatic metastases frequently show higher PD-L1 expression than lymph-node or peritoneal metastases. Third, PD-L1 expression is dynamically regulated. Chemotherapy, radiotherapy, biliary obstruction, and intercurrent infections can all upregulate PD-L1 through interferon-γ–dependent and independent pathways, meaning archival diagnostic tissue may not reflect the immune state at the time immunotherapy is initiated. Beyond assay-related issues, the BTC tumor microenvironment (TME) further erodes the predictive value of PD-L1. Dense desmoplasia, cancer-associated fibroblasts (CAFs), and abundant M2-polarized macrophages characterize the BTC TME (Loeuillard et al., 2020). In the cholangiocarcinoma microenvironment, bile acids activate the GPBAR1 receptor on CAFs, inducing CXCL10 secretion and neutrophil recruitment and thereby creating an immunosuppressive niche that can limit T-cell function irrespective of PD-L1 status (Greten et al., 2023). This biology may explain why some patients with PD-L1–negative tumors achieve durable responses while some with PD-L1–positive tumors do not. A 2025 retrospective analysis of 91 patients with BTC treated with chemotherapy plus immunotherapy found that PD-L1–negative patients had numerically longer median progression-free survival (9.9 vs. 4.8 months) and a higher ORR (27.6% vs. 15.9%) than PD-L1–positive patients (Torlakovic et al., 2020).

### 2.4 Emerging and composite biomarkers

Given the imperfect performance of individual conventional biomarkers, the field is moving toward multidimensional immune profiling and composite models to refine patient stratification for immunotherapy. Histopathologic features of the antitumor immune response provide complementary prognostic and predictive information. High densities of intratumoral CD8^+^ effector T cells and peritumoral tertiary lymphoid structures (TLS) are independently associated with improved survival in resected BTC (Brindley et al., 2021). In the metastatic setting, a 2023 multicenter retrospective study of 187 patients with BTC treated with checkpoint inhibitors showed that the presence of mature TLS was associated with a 2.4-fold higher ORR (38% vs. 16%) and a 48% reduction in the hazard of death (hazard ratio, 0.52; 95% CI, 0.33–0.82), even after adjustment for PD-L1 expression and TMB (Li et al., 2023). TLS serve as *in situ* sites for T-cell priming and B-cell affinity maturation, and their presence indicates an actively engaged adaptive immune response. However, standardized scoring criteria for TLS in BTC have not been established, and their predictive value in first-line chemoimmunotherapy has not been prospectively validated. Unlike static tissue biomarkers, ctDNA clearance during therapy provides a real-time, quantitative measure of therapeutic response. In advanced BTC treated with first-line chemoimmunotherapy, undetectable ctDNA after two to three cycles is associated with dramatically prolonged progression-free and overall survival, with hazard ratios for death ranging from 0.21 to 0.35 across retrospective cohorts (Liu et al., 2024). Notably, ctDNA response precedes radiologic response by 4–6 weeks, offering a potential window for early treatment adaptation. However, 15%–20% of patients with metastatic BTC have undetectable baseline ctDNA despite radiographically evident disease—particularly those with peritoneal-only metastases—limiting the universal applicability of this approach (Kato et al., 2024). Gut microbial composition modulates systemic immunity and checkpoint inhibitor efficacy across multiple cancers, and preliminary data extend this association to BTC. Responders to immunotherapy show higher baseline microbial diversity and enrichment of taxa such as *Bifidobacterium longum* and *Akkermansia muciniphila*, whereas antibiotic exposure within 30 days of treatment initiation is associated with a reduced ORR and shorter progression-free survival (Zhang et al., 2024). Interpretation in BTC is complicated by the high prevalence of biliary obstruction, recurrent cholangitis, and antibiotic exposure—all of which profoundly alter gut ecology independent of treatment response. Trials of fecal microbiota transplantation in BTC are under way, but current evidence remains preliminary. No single biomarker captures the full complexity of BTC immunobiology, and composite multi-feature models consistently outperform individual markers. For example, an “immune-inflamed” phenotype defined by combined PD-L1 expression, CD8^+^ T-cell density, and TLS presence identifies approximately 25% of patients with BTC who derive particularly favorable outcomes from chemoimmunotherapy, with 2-year overall survival rates exceeding 40% (Valle et al., 2017). Going forward, integrated models incorporating genomic alterations, transcriptomic immune signatures, spatial proteomics, and on-treatment ctDNA dynamics are likely to provide the most robust patient stratification. The prevalence, clinical utility, and core limitations of both classical and emerging biomarkers for BTC immunotherapy are comprehensively compared in Table 1.

**TABLE 1:** Established and emerging predictive biomarkers for immunotherapy in advanced biliary tract cancer.

Biomarker category	Specific biomarker	Prevalence in BTC	Predictive value for ICIs	Key limitations
Classical genomic biomarkers	dMMR/MSI-H	∼1–3% overall; DNA repair gene alterations detected in 13% ICC, 26% ECC, 6% GBC	Robust; ORR of 27%–37% in dMMR cohorts	Extremely low prevalence in unselected populations; requires standardized IHC and molecular testing
​	Tumor mutational burden (≥10 mut/Mb)	5%–8% overall; 2%–3% ultra-hypermutated (>20 mut/Mb)	Modest; clinical responses concentrated in the ultra-hypermutated subgroup	No BTC-specific cutoff consensus; high inter-assay variability; poor tissue-plasma concordance
​	PD-L1 expression	30% pooled positivity (range: 4%–76%)	Poor; no consistent predictive value across prospective trials	Antibody clone and scoring system heterogeneity; profound spatial tumor heterogeneity; dynamic expression regulation
Emerging tissue biomarkers	Mature tertiary lymphoid structures (TLS)	Variable	Strong; 2.4-fold higher ORR; 48% reduction in death hazard	No standardized BTC-specific scoring criteria; not prospectively validated in first-line chemoimmunotherapy
Dynamic liquid biomarkers	On-treatment ctDNA clearance	∼80% of patients have detectable baseline ctDNA	Strong; undetectable ctDNA after 2–3 cycles associated with dramatically prolonged PFS/OS	Not applicable in 15%–20% of patients (e.g., peritoneal-only metastases)
Host-related biomarkers	Gut microbiota composition	N/A	Preliminary; higher baseline diversity associated with improved response	Heavily confounded by biliary obstruction, cholangitis, and frequent antibiotic exposure
Composite models	Immune-inflamed phenotype (PD-L1 + CD8^+^ T cells + TLS)	∼25% of advanced BTC patients	Superior to single biomarkers; 2-year OS >40%	Retrospectively derived; lacks large-scale prospective validation

## 3 Early clinical trials of immune checkpoint inhibitors

The phase 1b KEYNOTE-028 trial provided the first proof-of-concept efficacy and safety data for PD-1 inhibition in PD-L1–positive advanced BTC (Piha-Paul et al., 2020). Of 89 screened patients, 37 (41.6%) had PD-L1–positive tumors (tumor-cell positivity ≥1%), and 24 were enrolled (20 with cholangiocarcinoma, 4 with GBC). The ORR was 17% (all partial responses), and an additional 17% of patients had stable disease, for a disease control rate of 34%. Median progression-free survival was 1.8 months (95% CI, 1.4–3.1), and median overall survival was 5.7 months (95% CI, 3.1–9.8). Median duration of response was not reached. Grade 3 treatment-related adverse events occurred in 21% of patients, with no grade 4 events or clinically significant hepatotoxicity (Piha-Paul et al., 2020). The larger phase 2 KEYNOTE-158 basket trial subsequently enrolled 104 patients with BTC whose disease had progressed after or who were intolerant to standard therapy, regardless of PD-L1 expression (Piha-Paul et al., 2020). Pembrolizumab monotherapy yielded an ORR of 5.8%, with a median duration of response that had not been reached (range, 6.2 to >26.6 months). Stable disease occurred in 17.3% of patients, for a disease control rate of 23.1%. Median progression-free survival was 2.0 months (95% CI, 1.9–2.1), and median overall survival was 7.4 months (95% CI, 5.5–9.6). Among patients with PD-L1–positive tumors (CPS ≥1), the ORR was 6.6%, as compared with 2.9% in those with PD-L1–negative tumors, a difference that did not reach statistical significance. Grade 3–5 treatment-related adverse events occurred in 13.5% of patients, including one grade 5 event of renal failure. A phase 1 study by Ueno and colleagues evaluated nivolumab in Japanese patients with unresectable or recurrent BTC, both as monotherapy and in combination with cisplatin plus gemcitabine (Ueno et al., 2019). In the monotherapy cohort (n = 30, previously treated), median progression-free survival was 1.4 months (90% CI, 1.4 to 1.4) and median overall survival was 5.2 months (90% CI, 4.5–8.7). In the combination cohort (n = 30, treatment-naïve), median progression-free survival was 4.2 months (90% CI, 2.8–5.6) and median overall survival was 15.4 months (90% CI, 11.8 to not estimable); the ORR was 37%, including one complete response (Ueno et al., 2019). Grade 3–4 adverse events occurred in 70% of the combination cohort, consistent with the safety profile of chemotherapy alone. Kim and colleagues reported a phase 2 multi-institutional trial of single-agent nivolumab in patients with advanced refractory BTC who had received at least one prior line of therapy (Kim et al., 2020). Among 46 evaluable patients, the confirmed ORR by independent central review was 11%, and an additional 38% had stable disease, for a disease control rate of 50%. All five responders had microsatellite-stable (MSS) tumors, indicating that MSS status does not preclude benefit. Median progression-free survival was 3.68 months (95% CI, 2.30–5.69), and median overall survival was 14.24 months (95% CI, 5.98 to not reached). Grade 3–4 treatment-related adverse events occurred in 17% of patients, with hyponatremia (6%) and increased alkaline phosphatase (4%) the most common. Several responders had prolonged disease control exceeding 12 months, suggesting that durable benefit is achievable in a subset of patients with MSS BTC despite the absence of classical biomarkers. The key efficacy and safety profiles of representative early-phase immune checkpoint inhibitor trials in advanced BTC are summarized in Table 2.

**TABLE 2:** Key early-phase clinical trials of immune checkpoint inhibitors in advanced biliary tract cancer.

Trial name	Phase	Intervention	Treatment line	N	ORR (%)	Median PFS (months)	Median OS (months)	Grade ≥3 TRAEs (%)
KEYNOTE-028	Ib	Pembrolizumab	≥2nd line (PD-L1 selected)	24	17.0	1.8	5.7	21.0
KEYNOTE-158	II	Pembrolizumab	≥2nd line (all-comer)	104	5.8	2.0	7.4	13.5
Ueno et al., 2019 (monotherapy cohort)	I	Nivolumab	≥2nd line	30	NR	1.4	5.2	NR
Ueno et al., 2019 (combination cohort)	I	Nivolumab + gemcitabine-cisplatin	1st line	30	37.0	4.2	15.4	70.0
Kim et al., 2020	II	Nivolumab	≥2nd line	46	11.0	3.68	14.24	17.0

## 4 Landmark phase 3 trials: chemoimmunotherapy as first-line standard

### 4.1 The TOPAZ-1 trial: durvalumab plus chemotherapy

The global, randomized, double-blind, placebo-controlled phase 3 TOPAZ-1 trial evaluated the addition of durvalumab, an anti–PD-L1 antibody, to gemcitabine and cisplatin in previously untreated patients with unresectable or metastatic BTC (Lamarca et al., 2024). Among 685 randomized patients, the durvalumab–chemotherapy arm showed a significant overall survival benefit at interim analysis (hazard ratio for death, 0.80; 95% CI, 0.66 to 0.97; P = 0.021). With updated follow-up (median, 23.4 months vs. 22.4 months), the hazard ratio further improved to 0.76. Overall survival rates at 12, 18, and 24 months all favored the durvalumab combination: 54.8% vs. 47.1%, 34.8% vs. 24.1%, and 23.6% vs. 11.5%, respectively. The ORR was 27% in the durvalumab arm vs. 19% in the control arm. Responders in the durvalumab arm had a median overall survival of 19.5 months, and more patients achieved survival of 18 months or longer (26% vs. 19%). The safety profile was consistent with the known profiles of each agent, with no new safety signals. The doubling of 24-month survival and the progressive improvement in the hazard ratio over time reflect a durable immunotherapy benefit, likely mediated by long-lasting T-cell memory. Real-world studies have corroborated these pivotal trial results. An international retrospective analysis of 666 patients across 39 centers in 11 countries reported a median overall survival of 15.1 months, median progression-free survival of 8.2 months, ORR of 32.6%, and disease control rate of 77.8% (Lamarca et al., 2024). An Italian real-world analysis of 145 patients found a median progression-free survival of 8.9 months, median overall survival of 12.9 months, grade 3–4 adverse events in 35.2% of patients, and immune-mediated events in 22.7% (Lamarca et al., 2022). Exploratory analyses from TOPAZ-1 indicated that PD-L1 expression, measured by the Tumor Area Positivity score, was not a reliable standalone predictor of benefit.

### 4.2 The KEYNOTE-966 trial: pembrolizumab plus chemotherapy

KEYNOTE-966 was a randomized, double-blind, placebo-controlled phase 3 trial evaluating pembrolizumab, an anti–PD-1 antibody, added to gemcitabine and cisplatin in 1,069 patients with previously untreated advanced BTC (Kelley et al., 2023). With a median follow-up of 25.6 months, pembrolizumab plus chemotherapy improved overall survival as compared with chemotherapy alone: median overall survival, 12.7 months (95% CI, 11.5–13.6) vs. 10.9 months (95% CI, 9.9–11.6); hazard ratio, 0.83 (95% CI, 0.72 to 0.95; one-sided P = 0.0034). After an additional 4 months of follow-up (median, 29.5 months), the overall survival benefit was sustained, with 24-month survival estimates of 24.7% vs. 19.1% (hazard ratio, 0.84; 95% CI, 0.74–0.96). In a Chinese subpopulation of 158 patients, pembrolizumab plus chemotherapy produced a median overall survival of 14.1 months, as compared with 9.9 months (hazard ratio, 0.74; 95% CI, 0.51–1.08), and 49% of responders maintained a response for 12 months or longer (Kelley et al., 2023). Grade 3–5 treatment-related adverse events occurred in 71.3% vs. 69.3% of patients; grade 3–5 immune-mediated events and infusion reactions occurred in 7.6% vs. 3.9% and were generally manageable.

### 4.3 The IMbrave151 trial: atezolizumab plus chemotherapy with or without bevacizumab

IMbrave151 (NCT04677504) is a global, randomized, double-blind, placebo-controlled phase 2 proof-of-concept trial evaluating the incremental benefit of adding bevacizumab to atezolizumab (anti–PD-L1) plus gemcitabine and cisplatin in previously untreated advanced BTC (Macarulla et al., 2025). A total of 162 patients were randomly assigned in a 1:1 ratio to receive atezolizumab (1,200 mg every 3 weeks) plus bevacizumab (15 mg/kg every 3 weeks) or matching placebo, in combination with cisplatin 25 mg/m^2^ and gemcitabine 1,000 mg/m^2^ on days 1 and 8 of each 21-day cycle. Chemotherapy was administered for up to 8 cycles, followed by maintenance atezolizumab with or without bevacizumab until disease progression or unacceptable toxicity. Stratification factors included geographic region and primary tumor location. The primary endpoint was investigator-assessed progression-free survival. At an updated analysis, median progression-free survival was 8.3 months in the bevacizumab arm and 7.9 months in the placebo arm (stratified hazard ratio, 0.67; 95% CI, 0.46–0.95), meeting the prespecified threshold for statistical significance in this signal-seeking study (Macarulla et al., 2025). No significant overall survival benefit was observed, however: median overall survival was 14.9 months in the bevacizumab arm vs. 14.6 months in the control arm (stratified hazard ratio, 0.97; 95% CI, 0.64–1.47). Objective response rates were nearly identical between arms (26.6% vs. 26.5%), but the median duration of response was longer with bevacizumab (10.3 vs. 6.2 months). Grade 3–4 treatment-related adverse events occurred in 74% of patients in both groups, with no new or unexpected safety signals. An exploratory biomarker analysis revealed that high tumoral *VEGFA* gene expression was associated with a greater progression-free survival benefit from the addition of bevacizumab (hazard ratio, 0.44; 95% CI, 0.23–0.83), suggesting that angiogenic biomarker stratification might identify patient subgroups that derive additional benefit from antiangiogenic plus chemoimmunotherapy (Macarulla et al., 2025). Of note, the control arm (atezolizumab plus chemotherapy alone) achieved a median overall survival of 14.6 months, which is numerically close to the immunotherapy–chemotherapy arms of TOPAZ-1 and KEYNOTE-966. Given the phase 2 design, smaller sample size, and differences in baseline patient characteristics and follow-up duration, this cross-trial comparison is provided for descriptive reference only and cannot support a conclusion of equivalent efficacy. To facilitate cross-trial comparison and contextualize design and population heterogeneity across first-line chemoimmunotherapy regimens, core study characteristics and clinical outcomes of the three landmark trials are summarized in Table 3.

**TABLE 3:** Pivotal phase 2/3 trials of first-line chemoimmunotherapy in advanced biliary tract cancer.

Trial name	Phase	Intervention arms	Total N	Primary endpoint	ORR (%)	Median PFS (mo)	Median OS (mo)	OS HR (95% CI)	Grade ≥3 TRAEs (%)
TOPAZ-1	III	Durvalumab + GCPlacebo + GC	685	Overall survival	2719	NR	NRNR	0.76 (0.66–0.97)	NR
KEYNOTE-966	III	Pembrolizumab + GCPlacebo + GC	1069	Overall survival	NRNR	NR	12.710.9	0.83 (0.72–0.95)	71.369.3
IMbrave151	II	Atezolizumab + bevacizumab + GCAtezolizumab + placebo + GC	162	Progression-free survival	26.626.5	8.37.9	14.914.6	0.97 (0.64–1.47)	7474

### 4.4 Clinical implications and critical appraisal

Together, the phase 3 TOPAZ-1 and KEYNOTE-966 trials establish chemoimmunotherapy as the first-line standard of care for advanced BTC, while the phase 2 IMbrave151 study provides complementary data on antiangiogenic intensification and atezolizumab-based regimens. Despite numerical differences in overall survival hazard ratios (0.76 for TOPAZ-1 vs. 0.83 for KEYNOTE-966), a matching-adjusted indirect comparison confirmed no significant efficacy gap between the durvalumab and pembrolizumab combinations, supporting comparable clinical benefit and underscoring the need for caution in unadjusted cross-trial comparisons. Heterogeneity in effect sizes across the three studies arises from key differences in design and population. IMbrave151 is a phase 2 signal-seeking trial with progression-free survival as its primary endpoint, limited statistical power for overall survival, and no pure chemotherapy control arm—factors that preclude direct estimation of the incremental overall survival benefit of PD-L1 inhibition (Kam et al., 2021). Chemotherapy protocols also differed: KEYNOTE-966 allowed investigator-discretionary continuation of gemcitabine beyond 8 cycles (43% of patients in the immunotherapy arm received ≥9 cycles), whereas the other two trials capped chemotherapy at 8 cycles, a difference that may influence both disease control and immune priming (Kelley et al., 2023). KEYNOTE-966 enrolled a higher proportion of non-Asian patients (55% vs. 45% in TOPAZ-1) with formal geographic stratification, and Asian subgroups show numerically greater overall survival benefit in both phase 3 trials (Lamarca et al., 2024). Distinct PD-L1 immunohistochemistry assays and scoring systems introduce analytical variability, and varying proportions of intrahepatic, extrahepatic, and gallbladder cancer subtypes may further shape outcomes, though all subgroup analyses are *post hoc* and hypothesis-generating only. Cross-trial subgroup analyses offer clinically relevant insights. Intrahepatic cholangiocarcinoma consistently shows the most pronounced overall survival benefit, likely driven by higher mutational burden and a more inflamed tumor microenvironment; other anatomic subtypes show more modest, nonsignificant improvements (Banales et al., 2020). Patients with baseline biliary obstruction or mild hyperbilirubinemia—representing 30%–40% of real-world cases but largely excluded from pivotal trials—derive a similar relative overall survival benefit yet face an elevated risk of hepatobiliary toxicity; expert consensus recommends pretreatment biliary drainage and optimization of liver function, though high-level supporting evidence is limited (Lamarca et al., 2022). All three first-line trials exhibit a characteristic dissociation between modest gains in progression-free survival (approximately 1.5–2 months) and more durable overall survival benefits with late-separating survival curves (Lamarca et al., 2024). Four BTC-specific mechanisms may explain this pattern: the delayed onset of checkpoint inhibitor activity; pseudoprogression in 3%–5% of patients, which artificially shortens RECIST 1.1–assessed progression-free survival; a unique first-line immunological window of opportunity that is not fully replicable in later treatment lines; and gradual remodeling of the dense desmoplastic stroma that facilitates effector T-cell infiltration over time. PD-L1 expression lacks consistent, statistically significant predictive value across the three studies. A numerical trend toward greater benefit in PD-L1–high subgroups in TOPAZ-1, together with well-documented challenges in assay standardization, suggests that refined, harmonized protocols could support clinical utility if prospectively validated (Lamarca et al., 2024). Despite the statistically significant overall survival benefit conferred by adding immune checkpoint inhibitors to first-line chemotherapy, the substantial economic burden of this approach must not be overlooked. The addition of durvalumab or pembrolizumab to the gemcitabine-cisplatin doublet is estimated to increase direct drug costs by nearly 70-fold, while the median progression-free survival gain is only approximately 1.5–2 months and the median overall survival gain approximately 1–2 months. This stark disparity between marginal clinical benefit and exponential cost escalation presents a major pharmacoeconomic challenge, particularly in resource-limited settings, and may exacerbate financial toxicity for patients and their families. This reality further underscores the urgent need to develop and validate robust predictive biomarkers to precisely identify the subset of patients most likely to derive durable benefit from this expensive therapy, thereby enabling more rational allocation of healthcare resources. In addition to the detailed summary of pivotal first-line trials in Table 3, we provide a comprehensive, line-by-line overview of benchmark chemotherapy, established immunotherapies, and investigational combinations across all treatment settings (first-line, second-line, and later-line) in Table 4, along with their key efficacy data and levels of evidence, to serve as a practical reference for clinical decision-making.

**TABLE 4:** Summary of benchmark chemotherapy and immunotherapy regimens for advanced biliary tract cancer.

Regimen category	Trial/Regimen	Phase	Intervention	Treatment line	Sample size (N)	ORR (%)	Median PFS (mo)	Median OS (mo)	Grade ≥3 TRAEs (%)
Benchmark chemotherapy (standard of care)	Gemcitabine + Cisplatin (historical reference)	III	Gemcitabine + cisplatin (GC)	1st-line	410	∼19–26	∼7.0–8.0	∼11.0–12.0	∼69
​	mFOLFOX (ABC-06)	III	Modified FOLFOX	2nd-line	162	NR	NR	6.2	NR
First-line chemoimmunotherapy (pivotal trials)	Durvalumab + GC (TOPAZ-1)	III	Durvalumab + gemcitabine + cisplatin	1st-line	685	27.0	NR	NR (HR 0.76 vs. control)	NR
​	Pembrolizumab + GC (KEYNOTE-966)	III	Pembrolizumab + gemcitabine + cisplatin	1st-line	1069	NR	NR	12.7	71.3
​	Atezolizumab + Bevacizumab + GC (IMbrave151)	II	Atezolizumab + bevacizumab + gemcitabine + cisplatin	1st-line	162	26.6	8.3	14.9	74.0
​	Atezolizumab + GC (IMbrave151 control arm)	II	Atezolizumab + gemcitabine + cisplatin	1st-line	162	26.5	7.9	14.6	74.0
First-line chemoimmunotherapy (exploratory trials)	Nivolumab + GC (Ueno et al., 2019)	I	Nivolumab + gemcitabine + cisplatin	1st-line	30	37.0	4.2	15.4	70.0
Second-/later-line ICI monotherapy	Pembrolizumab (KEYNOTE-028)	Ib	Pembrolizumab (PD-L1–selected)	≥2nd-line	24	17.0	1.8	5.7	21.0
​	Pembrolizumab (KEYNOTE-158)	II	Pembrolizumab (all-comers)	≥2nd-line	104	5.8	2.0	7.4	13.5
​	Nivolumab (Ueno et al., 2019)	I	Nivolumab	≥2nd-line	30	NR	1.4	5.2	NR
​	Nivolumab (Kim et al., 2020)	II	Nivolumab	≥2nd-line	46	11.0	3.68	14.24	17.0
Second-/later-line combination immunotherapy	Nivolumab + Ipilimumab (CheckMate 848, BTC subcohort)	II	Nivolumab + ipilimumab (high TMB population)	≥2nd-line	42	28.6	NR	NR (pending)	NR
​	Regorafenib + Nivolumab	Ib/II	Regorafenib + nivolumab	≥2nd-line	39	17.9	3.9	9.9	NR
​	Trastuzumab + Pembrolizumab	II	Trastuzumab + pembrolizumab (HER2-positive)	≥2nd-line	15 (preliminary)	33.3	NR	NR	NR

## 5 Expanded combination immunotherapy strategies

Although first-line chemoimmunotherapy has become the standard of care for advanced BTC, most patients will eventually experience disease progression and require subsequent therapy. In the second-line and later-line settings, the evidence for immunotherapy is derived primarily from early-phase trials, subgroup analyses of biomarker-selected populations, and exploratory studies of various combination strategies. Currently, later-line immunotherapy options include: (1) immune checkpoint inhibitor monotherapy (e.g., pembrolizumab or nivolumab), which shows limited efficacy in unselected patients but consistent benefit in those with dMMR/MSI-H or high TMB; (2) dual immune checkpoint blockade (nivolumab plus ipilimumab), primarily for patients with high TMB or dMMR/MSI-H; (3) immune checkpoint inhibitors combined with antiangiogenic multikinase inhibitors (e.g., regorafenib); and (4) immunotherapy combined with molecularly targeted agents (e.g., HER2-, FGFR2-, or IDH1-directed therapies) for patients harboring corresponding oncogenic drivers. Table 4 provides a systematic summary of treatment options, key efficacy data, and levels of evidence across all lines of therapy. The following subsections detail the clinical evidence and translational research progress for each of these combination strategies.

### 5.1 Immune checkpoint inhibitors plus antiangiogenic TKIs

Antiangiogenic agents normalize aberrant tumor vasculature, reduce interstitial fluid pressure, and enhance effector T-cell infiltration into the tumor parenchyma, providing a mechanistic rationale for synergy with immune checkpoint inhibitors (Galluzzi et al., 2020). Regorafenib, a multikinase inhibitor targeting VEGFR, TIE2, and oncogenic kinases, has been combined with immune checkpoint inhibitors in refractory BTC. A multicenter phase 1b/2 trial of regorafenib plus nivolumab enrolling 39 patients with previously treated BTC reported an ORR of 17.9% and a disease control rate of 64.1%, with a median progression-free survival of 3.9 months and a median overall survival of 9.9 months (Kang et al., 2023). Exploratory biomarker analyses suggested that patients with high angiogenic gene signatures derived greater progression-free survival benefit, supporting the hypothesis that antiangiogenic effects mediate the combinatorial synergy. Both regimens have manageable safety profiles in selected patients, but hepatobiliary toxicity remains the primary dose-limiting concern in those with compromised baseline liver function.

### 5.2 Immunotherapy combined with molecularly targeted agents

For BTC subtypes harboring actionable oncogenic drivers, combining targeted therapy with immunotherapy represents a precision immuno-oncology approach that simultaneously blocks oncogenic signaling and remodels the immune microenvironment. ERBB2 (HER2) amplification occurs in approximately 10%–15% of BTCs, with the highest prevalence in gallbladder cancer and extrahepatic cholangiocarcinoma (Javle et al., 2021). Preclinical data show that aberrant HER2 signaling upregulates PD-L1 expression and recruits immunosuppressive myeloid cells, providing a rationale for dual blockade. The MyPathway phase 2a basket trial evaluated trastuzumab plus pertuzumab in 39 patients with HER2-amplified or HER2-overexpressing BTC, yielding an ORR of 23% and durable responses exceeding 12 months in a subset of patients (Javle et al., 2021). Building on this, phase 2 trials of trastuzumab combined with PD-1 inhibitors are under way. Preliminary results from a single-arm phase 2 study of trastuzumab plus pembrolizumab in HER2-positive advanced BTC reported an ORR of 33.3% in the first 15 evaluable patients, with no unexpected safety signals (Lee et al., 2024). HER2-directed antibody–drug conjugates, such as trastuzumab deruxtecan, have also shown substantial activity in BTC, and their combination with immune checkpoint inhibitors is an active area of clinical investigation. FGFR2 fusions and IDH1 mutations are defining molecular subsets of intrahepatic cholangiocarcinoma, present in approximately 15% and 10%–20% of cases, respectively (Goyal et al., 2021). Historically, these alterations were associated with an immunologically “cold” TME and a reduced response to immune checkpoint inhibitors, but emerging preclinical evidence indicates that targeted inhibition can reverse immune suppression. For FGFR2 fusion–positive BTC, the phase 3 FIGHT-302 trial is evaluating pemigatinib in combination with pembrolizumab as first-line therapy. Preliminary data show an ORR of approximately 30%, with translational analyses demonstrating increased intratumoral CD8^+^ T-cell density and reduced myeloid-derived suppressor cell infiltration after treatment (Abou-Alfa et al., 2024). For IDH1-mutant cholangiocarcinoma, the oncometabolite R-2-hydroxyglutarate produced by mutant IDH1 epigenetically suppresses T-cell effector function; IDH1 inhibition can restore antigen presentation and T-cell infiltration. A phase 1b study of ivosidenib combined with atezolizumab reported a disease control rate of 52% and evidence of on-treatment immune activation, though response rates remain modest (Bridgewater et al., 2023). Definitive phase 3 data are lacking for both combinations, and they remain investigational outside clinical trials.

### 5.3 Dual immune checkpoint blockade

Combined blockade of PD-1 and CTLA-4 targets complementary steps in T-cell priming and activation, potentially generating stronger and more durable antitumor immunity than single-agent checkpoint inhibition. In BTC, dual immune checkpoint blockade has been evaluated primarily in the refractory setting and in biomarker-selected populations. In the CheckMate 848 basket trial, which enrolled patients with high-TMB solid tumors, the BTC subcohort (n = 42) showed that nivolumab plus ipilimumab achieved an ORR of 28.6%, as compared with 14.3% with nivolumab monotherapy, along with a trend toward improved progression-free survival (He et al., 2023). For patients with dMMR–MSI-H BTC, dual blockade is a treatment option supported by extrapolation from other gastrointestinal cancers, though BTC-specific prospective data are limited. A retrospective multicenter study of 28 patients with dMMR BTC found a numerically higher ORR with dual blockade (57% vs. 38% with monotherapy) but no statistically significant difference in overall survival, and higher rates of grade 3–4 adverse events (36% vs. 15%) (Marabelle et al., 2022). Dual blockade combined with stereotactic body radiotherapy (SBRT) is also under investigation, with the goal of leveraging immunogenic cell death and abscopal effects. A randomized phase 2 study of SBRT plus nivolumab with or without ipilimumab in metastatic BTC reported a clinical benefit rate of 31.0% in the triple-combination arm vs. 10.5% in the SBRT-plus-nivolumab arm, though overall survival did not differ significantly (Lamarca et al., 2022).

## 6 Cancer vaccines

Tumor antigens can elicit specific T-cell responses that promote tumor rejection, and therapeutic cancer vaccines deliver one or more such antigens to induce systemic antitumor immunity (Schumacher and Schreiber, 2015). Investigational vaccines in BTC include monoantigen peptide vaccines, multiantigen formulations, dendritic cell (DC)-based vaccines, and emerging personalized platforms. Wilms’ Tumor 1 (WT1) is expressed in 68%–80% of BTCs, and Mucin 1 (MUC1) in 44%–95%; both are associated with poor survival (Sakamoto et al., 2010). A trial of gemcitabine plus a WT1 peptide vaccine in eight patients with BTC showed stable disease lasting at least 2 months in four patients (Oka et al., 2020). A phase 1 study of a MUC1 vaccine in BTC or pancreatic cancer demonstrated safety and feasibility, with preliminary activity signals particularly in BTC (Sakamoto et al., 2010). Given heterogeneous antigen expression, multi-antigen vaccines may be more effective. Aruga and colleagues tested a vaccine targeting CDCA1, CDH3, and KIF20A in 9 patients; five had stable disease, and there were no grade 3 or 4 adverse events (Oka et al., 2020). Cell-based vaccines often elicit stronger cell-mediated immune responses. In a phase 1/2 trial of a MUC1-pulsed DC vaccine in 12 patients with BTC or pancreatic cancer, no notable adverse reactions were reported, and four patients remained disease-free without recurrence during 4 years of follow-up (Miyazawa et al., 2015). Although early-generation peptide and DC vaccines have established feasibility, their clinical efficacy has been limited by narrow antigen coverage, poor immunogenicity, and tumor immune escape. Next-generation vaccine platforms are now addressing these limitations. mRNA-based vaccines, which have shown transformative success in infectious diseases, are being developed for cancer immunotherapy. They enable rapid manufacturing, simultaneous delivery of multiple antigens, and intrinsic adjuvant activity through innate immune sensing. Personalized neoantigen vaccines, designed to target patient-specific somatic mutations, are particularly well suited to highly heterogeneous malignancies such as BTC because they bypass central immune tolerance and target unique tumor-specific epitopes. Preclinical models of cholangiocarcinoma have shown that neoantigen vaccines combined with checkpoint inhibitors induce robust antitumor T-cell responses and delay tumor growth. Several early-phase clinical trials of personalized neoantigen vaccines in BTC are under way, both as monotherapy and in combination with immune checkpoint inhibitors. Although early-generation peptide and DC vaccines have demonstrated feasibility and safety, their clinical efficacy has been limited by narrow antigen coverage, poor immunogenicity, and tumor immune escape. Next-generation platforms currently under investigation, such as mRNA-based and personalized neoantigen vaccines, may overcome some of these limitations; however, their clinical utility remains unproven and they are currently considered strictly investigational. Until data from larger, well-designed prospective clinical trials become available, cancer vaccines should be regarded as a research strategy rather than a clinically accessible immunotherapeutic option.

## 7 Adoptive cell therapy

### 7.1 CAR-T cell therapy

Chimeric antigen receptor (CAR) T-cell therapy has achieved transformative success in hematologic cancers, but its application in solid tumors—including BTC—faces barriers of target heterogeneity, limited tumor infiltration, and an immunosuppressive microenvironment. Several antigen targets are under active clinical investigation in BTC. Claudin 18.2 (CLDN18.2), a tight junction protein aberrantly expressed in 30%–40% of BTCs (particularly gastric-type cholangiocarcinoma), is the most advanced target to date. CT041 (satricabtagene autoleucel), an autologous CLDN18.2-targeted CAR-T product, was evaluated in a multicenter phase 1 trial enrolling patients with advanced gastrointestinal cancers, including a small BTC subcohort (Qi et al., 2023). Among evaluable heavily pretreated patients with BTC, the ORR reached 50.0%, with a median progression-free survival of 4.4 months and a manageable safety profile dominated by grade 1–2 cytokine release syndrome. This represents the first CAR-T therapy to demonstrate meaningful clinical activity in BTC; however, these data derive from a small phase 1 subcohort and require confirmation in larger prospective trials. Phase 2 confirmatory studies are ongoing. Glypican-3 (GPC3) is highly expressed in hepatocellular carcinoma and in a subset of intrahepatic cholangiocarcinoma. GPC3-targeted CAR-T cells armored with a dominant-negative TGF-β receptor have shown promising activity in hepatocellular carcinoma (Lu et al., 2024), and early-phase basket trials are now enrolling patients with GPC3-positive BTC to evaluate safety and preliminary efficacy. Additional targets under preclinical and early clinical development include HER2, mesothelin, and CEA, but BTC-specific clinical data remain very limited. It is crucial to emphasize that CAR-T cell therapy in BTC remains in its earliest stages of exploration. The promising but preliminary results described above derive from small phase 1 subcohorts with extremely limited sample sizes, and the clinical benefit is modest and not yet definitive. CAR-T therapy in solid tumors faces fundamental barriers—including target antigen heterogeneity, poor tumor infiltration, an immunosuppressive microenvironment, and on-target off-tumor toxicity—that are particularly pronounced in BTC. Therefore, CAR-T cell therapy remains far from becoming a standard of care for BTC, and its clinical application should be strictly confined to clinical trials.

### 7.2 Tumor-infiltrating lymphocyte therapy

Tumor-infiltrating lymphocyte (TIL) therapy isolates autologous T cells from resected tumor tissue, expands them *ex vivo* to large numbers, and reinfuses them after lymphodepleting chemotherapy. TIL therapy has yielded durable complete responses in melanoma and cervical cancer, and its application in BTC is being actively explored. A novel engineered TIL product (ScTIL), derived from peripheral PD-1^+^ circulating T cells and modified with an enhanced PD-L1/CD28 co-stimulatory receptor, was evaluated in a phase 1 study of 10 patients with advanced BTC (Wang et al., 2024). At the recommended dose level, monotherapy achieved a median overall survival of 18.3 months, with no severe cytokine release syndrome or neurotoxicity; notably, this product does not require conventional high-dose chemotherapy preconditioning, which improves tolerability. Given the extremely small sample size and phase 1 design, these efficacy results are preliminary and hypothesis-generating only. Conventional autologous TIL therapy is also being investigated in BTC within clinical trials, and early case reports describe durable complete responses in patients with mutation-specific, neoantigen-reactive T cells (Tran et al., 2014). Widespread application, however, is limited by the requirement for accessible tumor tissue, complex manufacturing logistics, and the dense desmoplastic stroma of BTC, which restricts endogenous T-cell infiltration.

### 7.3 Core challenges for cell therapy in BTC

Despite encouraging early signals, several fundamental challenges limit the broader clinical application of cell therapy in BTC. First, intratumoral antigen heterogeneity leads to antigen escape and treatment failure; no single antigen is uniformly expressed across all tumor cells in BTC, and clonal selection under therapeutic pressure frequently results in the outgrowth of antigen-negative subclones. Second, the dense desmoplastic stroma and abundant immunosuppressive myeloid populations create physical and biochemical barriers that prevent infused T cells from trafficking into and persisting within tumor nests. High interstitial fluid pressure and aberrant vasculature in BTC further impair T-cell homing. Third, on-target, off-tumor toxicity is a critical concern: targets such as CLDN18.2 are expressed at low levels in normal gastric and biliary epithelium, carrying risks of mucosal damage and biliary epithelial injury. Finally, autologous cell products require individualized manufacturing with turnaround times of 2–4 weeks, which is challenging for patients with rapidly progressive advanced BTC. Ongoing strategies to address these barriers include armored CAR-T designs that resist TGF-β suppression, allogeneic “off-the-shelf” cell products, and combination with stroma-modulating agents to improve T-cell penetration.

## 8 Safety and management of immune-related adverse events in BTC

### 8.1 Incidence and spectrum of hepatobiliary irAEs

In the phase 3 first-line chemoimmunotherapy trials, grade 3–4 liver enzyme elevations occur in approximately 10%–15% of patients, a rate slightly higher than with chemotherapy alone. Immune-mediated hepatitis typically presents with predominant alanine aminotransferase (ALT) or aspartate aminotransferase (AST) elevation and usually manifests within the first 3 months of treatment. Immune-related sclerosing cholangitis is a rare but BTC-specific immune-related adverse event that occurs more frequently in patients with biliary tract malignancies than in those with other tumor types. It manifests with predominant elevations of alkaline phosphatase and γ-glutamyl transferase, often accompanied by hyperbilirubinemia and pruritus. Cross-sectional imaging may show biliary strictures and diffuse wall thickening, which can be radiologically indistinguishable from tumor progression or benign strictures related to prior stenting. The estimated incidence is 1%–3% in patients receiving anti–PD-1 or anti–PD-L1 monotherapy and up to 5% with dual checkpoint blockade.

### 8.2 Differential diagnosis and diagnostic evaluation

Diagnosis of hepatobiliary immune-related adverse events requires careful exclusion of alternative causes that are highly prevalent in the BTC population: tumor progression causing extrinsic biliary compression or hepatic parenchymal infiltration; ascending cholangitis secondary to indwelling biliary stents or obstruction; drug-induced liver injury from chemotherapy, analgesics, or supportive medications; reactivation of chronic viral hepatitis (HBV, HCV); and underlying nonalcoholic steatohepatitis or cirrhosis. The recommended diagnostic workup includes detailed liver function testing (fractionated bilirubin, ALT, AST, alkaline phosphatase, and γ-glutamyl transferase), viral hepatitis serology, abdominal cross-sectional imaging with cholangiographic sequences, and liver biopsy for diagnostically ambiguous cases. Distinguishing immune-related sclerosing cholangitis from mechanical obstruction is of particular clinical importance, because the two conditions demand fundamentally different management strategies.

### 8.3 Management principles and clinical practice recommendations

Management of hepatobiliary immune-related adverse events follows the general grading frameworks established by ESMO and ASCO clinical practice guidelines for immune-related toxicities but is tailored to the hepatobiliary context. For grade 1 hepatotoxicity (ALT or AST <3 times the upper limit of normal and alkaline phosphatase <2.5 times the upper limit of normal), immunotherapy may be continued with close weekly monitoring of liver function, systematic evaluation for secondary causes, and optimized biliary drainage if clinically indicated (Schneider et al., 2021). For grade 2 hepatotoxicity, immunotherapy should be withheld, oral corticosteroids at 0.5–1 mg/kg/day prednisone equivalent should be initiated after excluding infection and mechanical obstruction, and ursodeoxycholic acid (13–15 mg/kg/day) should be added for cholestatic-predominant injury, with liver biochemistry monitored twice weekly. Rechallenge with immunotherapy may be considered after a gradual steroid taper once biochemical values return to grade 1. For grade 3–4 hepatotoxicity, immunotherapy must be permanently discontinued; intravenous methylprednisolone at 1–2 mg/kg/day should be administered. In steroid-refractory cases persisting beyond 48–72 h, mycophenolate mofetil or tacrolimus may be added, with urgent hepatology consultation. For confirmed immune-related sclerosing cholangitis, endoscopic evaluation and temporary biliary stenting may be required alongside systemic immunosuppression. For patients with baseline biliary obstruction, expert consensus recommends adequate biliary drainage and optimization of liver function before initiating immunotherapy. Patients with indwelling biliary stents require close monitoring for ascending cholangitis; routine prophylactic antibiotics are not recommended, but prompt broad-spectrum antibiotic therapy is essential for suspected cholangitis.

## 9 Future developments and ongoing challenges

### 9.1 Future developments

**FIGURE 1:**
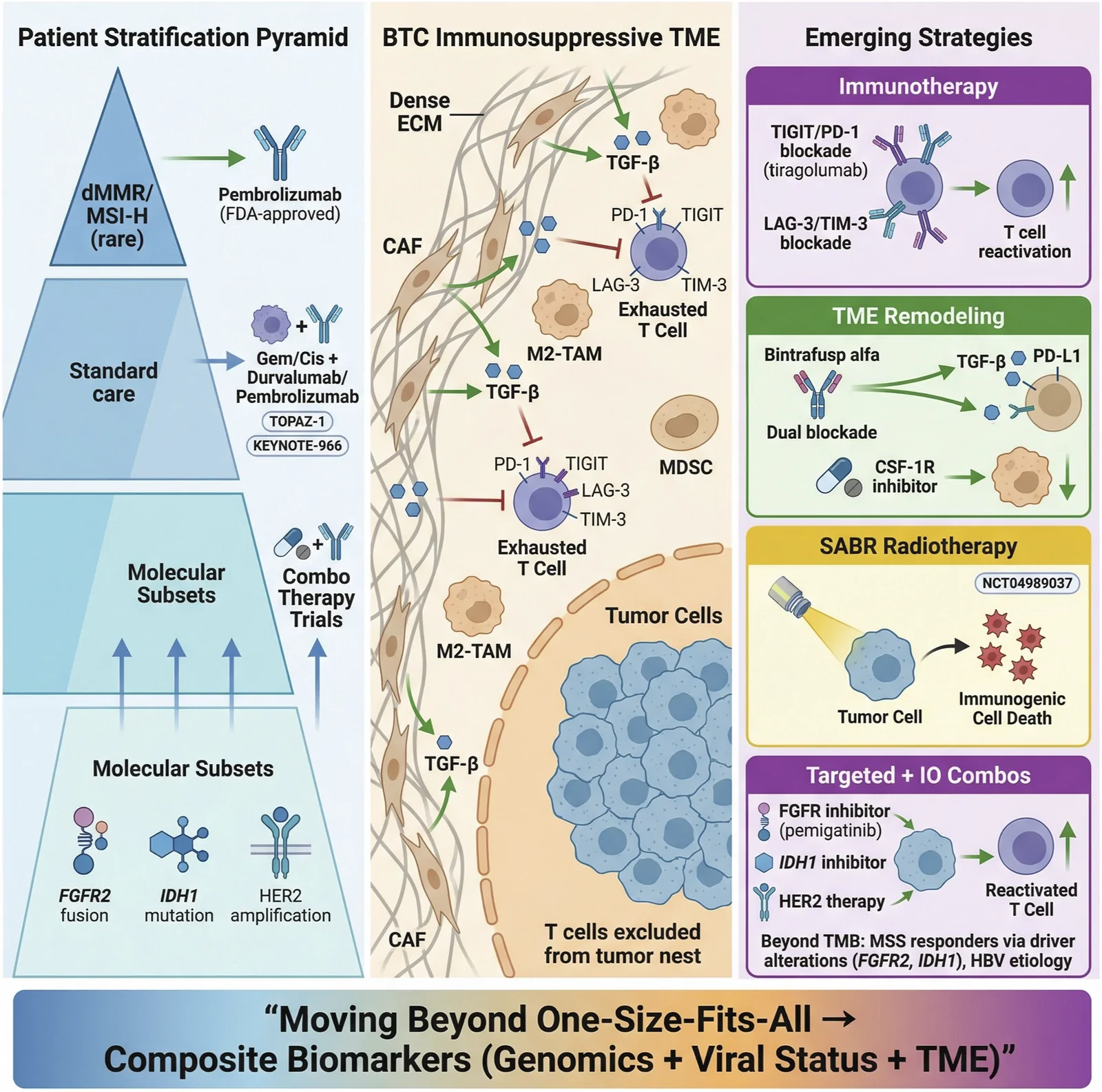
Precision immunotherapy paradigm and future directions in advanced biliary tract cancer.

The treatment of advanced BTC is evolving toward precision strategies centered on three pillars: refined biomarker stratification, mechanistic characterization of the tumor microenvironment, and rationally designed novel combination regimens. Key developmental directions include exploring the potential of next-generation checkpoint targets (e.g., LAG-3 and TIGIT) to reverse T-cell exhaustion; however, it is important to note that recent phase 3 trials across multiple solid tumors (including those targeting the TIGIT and LAG-3 pathways) have failed to demonstrate significant incremental benefit over PD-1/PD-L1 blockade alone, and their clinical value in BTC has been substantially de-validated. Consequently, future research should adopt a more cautious approach in evaluating these targets and redirect focus toward more promising TME-remodeling strategies, such as dual TGF-β/PD-L1 blockade, myeloid-cell modulators (CSF-1R inhibitors, CD40 agonists), and stroma-normalizing approaches to enhance T-cell infiltration; SBRT combined with immunotherapy to induce immunogenic cell death; and biomarker-driven combinations of immune checkpoint inhibitors with FGFR or IDH1-targeted agents to convert immunologically ‘cold’ tumors to ‘hot’ ones. Composite biomarker models integrating driver mutations, viral etiology, and TME features are under development, drawing strategic insights from more mature stratification frameworks in gastric and gastroesophageal junction cancer. Collectively, BTC immunotherapy is moving beyond one-size-fits-all chemoimmunotherapy toward personalized precision regimens built on multi-omics stratification and TME-modulating combinatorial approaches. Figure 1 visualizes an integrated framework covering hierarchical patient stratification, core immunosuppressive features of the BTC TME, and the full spectrum of emerging therapeutic pipelines. This schematic highlights two major barriers to durable immunotherapy efficacy: heterogeneous patient subgroups with inadequate standalone biomarkers and a desmoplastic, immune-suppressive TME that limits T-cell function. We further dissect the unmet translational challenges of these novel strategies in the following section.

### 9.2 Ongoing challenges

Several fundamental challenges continue to limit progress in BTC immunotherapy. Multi-checkpoint combination regimens carry an elevated risk of immune-related toxicity, a particular concern given the baseline hepatic vulnerability and biliary dysfunction common in patients with BTC. The desmoplastic, immune-excluded TME forms a robust barrier to T-cell trafficking and effector function, and current stroma-remodeling strategies still lack confirmatory phase 3 evidence. Classical genomic biomarkers (dMMR/MSI, TMB) have low prevalence in unselected BTC, and PD-L1 remains an unreliable predictor because of assay inconsistency and profound spatial tumor heterogeneity. Inter- and intratumoral molecular heterogeneity drives both primary and acquired resistance, and the mechanisms underlying durable responses in microsatellite-stable, low-TMB BTC remain poorly defined. Novel therapeutic modalities—including CAR-T cells, personalized vaccines, and TIL therapy—face shared translational bottlenecks: target antigen heterogeneity, on-target off-tumor toxicity, manufacturing complexity, and limited tumor infiltration of infused cells.

## 10 Conclusion

The phase 3 TOPAZ-1 and KEYNOTE-966 trials have established chemoimmunotherapy as the new first-line standard of care for advanced BTC, marking the field’s first major therapeutic advance in more than a decade, with meaningful improvements in overall survival and manageable safety profiles. Critical challenges remain, however: primary and acquired resistance driven by the profoundly immunosuppressive, desmoplastic tumor microenvironment; limited single-agent immunotherapy activity in unselected populations; the absence of robust, validated predictive biomarkers; and suboptimal management of hepatobiliary immune-related adverse events. Looking ahead, key research priorities include dissecting the immunobiology of the BTC tumor microenvironment, developing clinically validated composite biomarker panels, evaluating novel combinations that target alternative checkpoints and stromal or myeloid components, and advancing cellular therapies and personalized vaccines. These efforts aim to expand durable clinical benefit and shift the treatment paradigm from uniform, one-size-fits-all regimens toward precision-based care tailored to individual patients with BTC.
